# Correlation between pre-treatment quasispecies complexity and treatment outcome in chronic HCV genotype 3a

**DOI:** 10.1186/1743-422X-5-78

**Published:** 2008-07-09

**Authors:** Isabelle Moreau, John Levis, Orla Crosbie, Elizabeth Kenny-Walsh, Liam J Fanning

**Affiliations:** 1 Molecular Virology Diagnostic & Research Laboratory, Department of Medicine, Clinical Sciences Building, Cork University Hospital, Cork, Ireland; 2 Department of Gastroenterology, Cork University Hospital, Cork, Ireland

## Abstract

Pre-treatment HCV quasispecies complexity and diversity may predict response to interferon based anti-viral therapy. The objective of this study was to retrospectively (1) examine temporal changes in quasispecies prior to the start of therapy and (2) investigate extensively quasispecies evolution in a group of 10 chronically infected patients with genotype 3a, treated with pegylated α2a-Interferon and ribavirin.

The degree of sequence heterogeneity within the hypervariable region 1 was assessed by analyzing 20–30 individual clones in serial serum samples. Genetic parameters, including amino acid Shannon entropy, Hamming distance and genetic distance were calculated for each sample. Treatment outcome was divided into (1) sustained virological responders (SVR) and (2) treatment failure (TF).

Our results indicate, (1) quasispecies complexity and diversity are lower in the SVR group, (2) quasispecies vary temporally and (3) genetic heterogeneity at baseline can be use to predict treatment outcome.

We discuss the results from the perspective of replicative homeostasis.

## Background

The Hepatitis C virus (HCV), is the causative agent of chronic hepatitis C and infects at least 170 million individuals worldwide [1-3]. The virus has been classified into six major genotypes and more than 70 subtypes based on sequence diversity [4-10]. The most important feature of HCV is that it causes chronic infection in about 50–80% of individuals [3,11-13].

The HCV genome exhibits significant genetic heterogeneity due to accumulation of mutations during viral replication, attributed to a limited fidelity of the RNA dependent RNA polymerase [14,15]. This phenomenon generates a dynamic population of heterogeneous but closely related variants designated as quasispecies [14-17]. The massive genetic heterogeneity present in quasispecies has important biological consequences and enables HCV to escape immune clearance and to establish chronic infection [18-22]. Furthermore, the quasispecies distribution may influence the outcome of anti-viral therapy and be important in the development of resistance to anti-viral therapy [23-27]. It is well established that HCV genotype influences both response to therapy and disease severity as well as the viral-host interactions [19,28-30]. Patients infected with HCV genotypes 2 or 3 respond more favourably than genotype 1 to pegylated α2a-Interferon and ribavirin anti-viral therapy [12,27,31,32].

The HCV genomic heterogeneity is not distributed evenly across the HCV genome. In particular, the untranslated region at 5' and 3' ends of the genome exhibits areas of conservations, whereas the hypervariable region 1 (HVR1) located in the amino-terminus of the HCV envelope glycoprotein E2 is the most variable part of the HCV genome. There is strong evidence to suggest that the HVR1, encoding 27 amino acids (positions 1491 to 1571 on reference strain H77), is susceptible to immune pressure involving neutralising antibodies and allows the selection of escape mutants [27,31,33-36]. A considerable number of investigations into HCV quasispecies have focused on the analysis of the HVR1, given that a high degree of diversity increases the likelihood of distinguishing one viral species from another. Many studies have investigated the composition and the evolution of HCV quasispecies to determine whether the genetic changes could provide biological clues for understanding and predicting the outcome of anti-viral therapy. These studies have suggested a correlation between a high level of heterogeneity within the HVR1 and a poor response to pegylated α2a-Interferon and ribavirin therapy [21,28,30,31,37-43].

A growing body of evidence suggests that the molecular profile of an individual's pre-treatment HCV quasispecies diversity (QD) could potentially be used to identify responders and non-responders. Currently there is little information on the temporal changes to the QD in chronic HCV carriers prior to therapy as QD is usually assessed only at baseline [28,30,37-41,43]. Mapping sequential alteration to the QD may define possible windows of opportunity during which therapy may have increased efficacy for patients.

A mechanistic explanation for the temporal patterns of quasispecies complexity in the non treatment period may be found in replicative homeostasis (RH), a recently proposed hypothesis [44-47]. Briefly, RH consists of a series of autoregulatory feedback epicycles that link RNA polymerase function, RNA replication and protein synthesis through interactions between mutant or wild type proteins and the RNA dependant RNA polymerase (RDRP) causing formation of stable, but reactive, replicative equilibria [47]. Replicative homeostasis provides a rational explanation for HCV persistence, for HCV viral kinetics, for quasispecies stability and also for the various responses seen during anti-viral treatment of HCV. Recently Chen et al. have reported a study on Hepatitis B virus (HBV) which provides solid experimental evidence of replicative homeostasis [48]. The authors have demonstrated that mutant pre-core protein significantly reduces HBV replication and HBe antigen (HBeAg) expression relative to the wild type protein [48].

In the present study we have retrospectively investigated the genetic distance profile and the molecular evolution of the HCV quasispecies of a group of patients chronically infected with HCV genotype 3a (1) in the pre-treatment period and (2) during the course of treatment with pegylated α2a interferon plus ribavirin. Our goals were to define (1) temporal changes in QD during the time prior to therapy and (2) whether the patterns of these changes would correlate with the outcome of anti-viral therapy.

## Results

### Characterisation of the study group

All the samples used in this study were obtained from chronically infected patients with genotype 3a hepatitis C virus. The total number of individuals was 10; n = 7 females. Patient demographic details are outlined in Table 1. 9/10 patients were treatment naïve prior to the start of the standard 24 weeks pegylated α-2a interferon plus ribavirin therapy (Table 1). Among the ten patients included in the study, 6 were classified as sustained virological responders, hence SVR, and 4 were classified as treatment failure, hence TF (Table 1). Within the SVR group one patient, SVR12, could be classified as superfast responder, hence SFR, as HCV RNA was undetectable in serum at week 1 of treatment (Table 1). Within the TF group, one patient was classified as a non responder, hence NR2, as the viraemia remained stable during the whole course of treatment, whereas the three others were classified as relapsers, hence R (Table 1). A t-test was performed to investigate whether factors as age, body mass index (BMI) and Viral load at baseline and were significantly different between SVR and TF group. None of these comparison were significantly different (P > 0.05, data not shown). [49,50].

**Table 1:** Demographic details, treatment outcomes based on virologic responses, viral load at baseline and serial serum samples analysed over time for HCV genotype 3a chronically infected patients

**Patient Group**	**Type of Response**	**Sex**	**Rx Naïve**	**Age (years) at Baseline**	**Viral Load log_10 _IU/ml at Baseline**	**Time points**						
						
						**Pre treatment period**		**Early treatment period**				**Post treatment period**
**Sustained virological response (SVR)**				**Mean Age 41 ± 12**	**Mean VL 5.66 ± 0.66**	**E**	**B**	**W1**	**W2**	**W3**	**W4**	**L**

SVR3	SVR	F	Yes	28	5.47	+	+	+	+	+	- (V)	TND
SVR6	SVR	F	Yes	35	5.16	+	+	- (V)	TND	TND	TND	TND
SVR7	SVR	F	Yes	32	5.46	+	+	+	- (V)	TND	TND	TND
SVR8	SVR	F	Yes	59	6.89	+	+	+	NA	NA	+ (V)	TND
SVR9	SVR	F	No	45	6.37	+	+	+	+	NA	- (V)	TND
SVR12	SFR	F	Yes	49	5.17	+	+	TND	TND	TND	TND	TND

**Treatment failure (TF)**				**Mean Age 41 ± 7**	**Mean VL 6.23 ± 0.63**	**E**	**B**	**W1**	**W2**	**W4**	W12*	**L**

NR2	NR	F	Yes	42	5.05	+	+	+	NA	+	+	+ (W3)
R1	R	M	Yes	46	7.5	+	+	+ (V)	TND	TND	TND	+ (W2)
R4	R	M	Yes	45	7.11	+	+	+	+ (V)	TND	TND	+ (W10)
R13	R	M	Yes	31	6.32	+	+	+	-	TND	TND	- (W12)

The pre treatment period corresponds to E and B time point. E for early sample, taken between 6 to 12 months before treatment and B for baseline sample, taken at day 0 of pegylated INF-α2a/ribavirin treatment. The early treatment period corresponds to W1 to W4 time points (samples taken at 1, 2, 3 or 4 weeks of treatment). The sample taken at week 12 of treatment was only available for the non-responder patient (W12*). The post treatment period corresponds to the L time point and was only available within the TF group. L for late sample taken at 2, 3, 10 or 12 weeks after the end of treatment). **+**, sample available with successful analysis. **-**, sample available with unsuccessful analysis. TND, target not detected when HCV RNA was not detectable in the sample. (V), sample treated with the Viraffinity™ reagent. NA, sample non available for analysis.

### Clonal analysis and sequences data

Reproducibility, accuracy and sensitivity of the RT-PCR protocol were assessed by use of sera normalised to 4 log_10 _IU/mL and by use of *Pwo *DNA polymerase which exhibits proofreading activity [51].

In the present study, between 2 and 6 serial samples per individual were subjected to RT-PCR and clonal sequence analysis with a mean of 23 individual clones sequenced for each serum sample (Table 1). A total number of 839 molecular clones were recovered. The sequence analysis was performed after exclusion of all the defective sequences: nucleotide deletion (n = 2) or mutation (n = 3) producing a stop codon. A total number of 834 molecular clones, corresponding to a total of 267240 bp, were further examined. Sequence analysis of these 834 individual clones revealed a sequence of 320 bp in length encompassing the 81 bp of the HVR1, except for 30 clones which presented with a 12 nucleotide in-frame insertion. No other insertions were observed among the entire clonal population. For the purpose of the genetic analysis, the 804 sequences consisting of a 320 bp amplicon and the 30 sequences consisting of 332 bp amplicon (12 bp insertion) were trimmed by 14 bp, (specifically, 9 bp at the 5'end and 5 bp at the 3'end of the amplicon) leading to a final sequence of 306 bp or 318 bp (12 bp insertion), respectively. The 834 trimmed sequences were assigned unique GenBank accession nos. EU023073–EU023906. The 12 bp insertion observed among 30 individual molecular clones, is located exactly at the junction of the E1 and E2 regions (5'end of the 27 aa HVR1) and encoded a sequence of 4 aa. All of the 30 individual clones belonged to patient SVR6. A description of the molecular clones containing the 12 bp insertion is detailed at the end of the results section.

Phylogenetic trees reconstruction has shown independent clustering of the sequences from each individual or set of separate sequences. This finding confirms the absence of inter sample contamination (data not shown).

### Genetic variation during the pre-treatment assessment period

**Figure 1:**
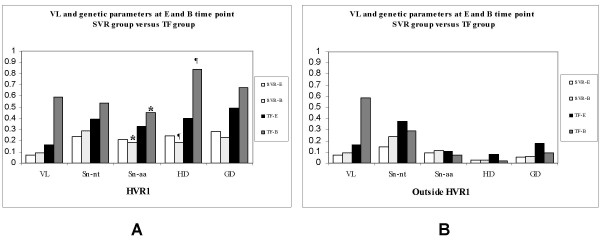
**Viral Load and genetic parameters in the two groups of patient (SVR and TF group) and at two time points (E, prior therapy and B, at baseline)**. In order to provide a mean value for multi parameter comparison, the variables were adjusted to fit to an appropriate scale i.e, (VL) Serum HCV RNA, No. of copies/ml × by a factor of 2.10^-8^, (Sn-nt) normalised entropy at nucleotide level and (Sn-aa) at amino acid level are actual values, (HD) mean Hamming distance × by a factor of 5 and (GD) genetic distance × by a factor of 10. The genetic parameters (Sn-nt, Sn-aa, HD and GD) were calculated (A), within the HVR1 (27 aa) and (B), outside the HVR1 (62 aa). (*), *P *= 0.019 for Sn-aa and (¶), *P *= 0.019 for HD, represent significant difference between the SVR and the TF group at B time point calculated by non parametric Mann-Whitney U test.

A serum sample 24–44 weeks prior to the start of therapy was available for each patient. This early sample, hence E, represents an intra-patient untreated control. The mean time between the E sample and the baseline sample, hence B, was 34 weeks (SEM ± 10) for the SVR group and 24 weeks (SEM ± 0) for the TF group (Table 2). At E and B time points the viral load did not differ significantly among SVR and TF groups (*P *> 0.05, Figure 1A). The changes in viral load observed for E vs B time points and B vs W1 time points were found to be significant within each group of patient but were non significant for inter-group comparison (Table 2). Although at E and B time points, within the HVR1, samples from the TF group exhibited higher viral load and higher quasispecies complexity (QC) than patients in the SVR group, (1) the viral load, (2) the normalised Shannon entropy at the nucleotide level (Sn-nt) and (3) the genetic distance (GD) did not differ significantly between the two groups of patients (*p *> 0.05, Figure 1A). In contrast, the normalised Shannon entropy at the amino acids level (Sn-aa) and the genetic diversity (mean Hamming distance, HD), within the HVR1, were significantly lower in the SVR than in the TF group at B time point (*P *= 0.019 for both parameters, Figure 1A) but not at E time point (*P *> 0.05, Figure 1A). The same analysis was performed on the 62 predicted aa sequences outside the HVR1 located at the 5'end of the HVR1. In all patient groups, the normalized Shannon entropy at both nucleotide and amino acid level, the genetic diversity and the genetic distance were always lower outside the HVR1 than within the HVR1 (Figure 1B). No significant difference for any of the genetic parameters examined outside the HVR1 was observed at E or at B time point between the two groups of patients (*P *> 0.05, Figure 1B).

**Table 2:** Changes within HVR1 and outside HVR1 in viral load, normalised entropy, genetic diversity and genetic distance in patients with chronic hepatitis C according to their response to pegylated α2a-interferon/ribavirin therapy

	**Patient group**	**No. of patients**	**Time points**	**Interval mean weeks**	**Change in serum HCV RNA × 10**^5 ^**copy/ml**	**Change in Normalised Shannon Entropy (Nucleotides)**	**Change in Normalised Shannon Entropy (Amino Acids)**	**Change in genetic diversity (mean Hamming distance)**	**Change in genetic distance**
	**SVR**	**6**	**E vs B**	34 ± 10	**3.56 ± 9.01†**	0.030 ± 0.297	-0.026 ± 0.266	-1.15 ± 4.56	-0.005 ± 0.025
		**4**	**B vs w1**	1 ± 0	**-17.21 ± 26.33†**	-0.177 ± 0.329	-0.111 ± 0.225*	-0.05 ± 4.14	0.002 ± 0.026
		**2**	**B vs w2**	2 ± 0	-25.98 ± 30.95	-0.001 ± 0.267	-0.008 ± 0.257	1.10 ± 1.00	-0.001 ± 0.004
		**2**	**B vs W3/4**	3.5 ± 0.5	-39.47 ± 38.11	-0.243 ± 0.064	-0.178 ± 0.156	-3.35 ± 1.85	-0.027 ± 0.015

**HVR1**									

	**TF**	**4**	**E vs B**	24 ± 0	**83.31 ± 92.20‡**	0.147 ± 0.063	0.118 ± 0.141	8.75 ± 4.13	0.018 ± 0.032
		**4**	**B vs w1**	1 ± 0	**-116.22 ± 125.37‡**	-0.126 ± 0.236	-0.086 ± 0.146*	-7.63 ± 9.98	-0.026 ± 0.036
		**2**	**B vs W2/4**	3 ± 1	-49.94 ± 56.35	-0.073 ± 0.268	-0.049 ± 0.220	-0.50 ± 3.70	-0.001 ± 0.016
		**3**	**B vs L**	5 ± 4	-33.70 ± 48.92	-0.336 ± 0.348	-0.398 ± 0.172	-15.60 ± 15.99	-0.054 ± 0.056

	**SVR**	**6**	**E vs B**			0.092 ± 0.232	0.024 ± 0.174	0.02 ± 0.77	0.002 ± 0.006
		**4**	**B vs w1**			-0.104 ± 0.108	-0.049 ± 0.142	-0.45 ± 0.62	-0.004 ± 0.005
		**2**	**B vs w2**			0.127 ± 0.047	-0.003 ± 0.054	0.05 ± 0.05	0.000 ± 0.001
		**2**	**B vs W3/4**			-0.071 ± 0.185	-0.002 ± 0.026	-1.35 ± 0.95	-0.013 ± 0.010

**Outside**									

	**TF**	**4**	**E vs B**			-0.089 ± 0.276	-0.035 ± 0.223	0.18 ± 1.43	-0.010 ± 0.018
		**4**	**B vs w1**			0.064 ± 0.274	0.009 ± 0.065	0.15 ± 0.43	-0.005 ± 0.008
		**2**	**B vs W2/4**			0.106 ± 0.239	0.032 ± 0.049	0.10 ± 0.10	0.001 ± 0.003
		**3**	**B vs L**			-0.007 ± 0.109	-0.049 ± 0.148	0.12 ± 0.48	-0.007 ± 0.009

SVR correspond to the sustained virological response patient group. TF correspond to the treatment failure group. The number of patients indicates the number of samples available for analysis at the corresponding time points. E represents the early time point, B the baseline or day 0 of treatment, W1–4 the week 1 to week 4 of treatment and L the sample taken after the end of treatment only available for analysis in the TF group. Negative values correspond to a reduction in HCV RNA level, normalized entropy at nucleotides or amino acids level, mean Hamming distance and genetic distance. The data represent mean ± SEM. The statistical significance of comparisons between time points and between the two groups of patients were analysed with non parametric Mann-Whitney U test. †, *P *= 0.01 for the change between time point E vs B and B vs W1 within the SVR group. ‡, *P *= 0.057 for the change between time point E vs B and B vs W1 within the TF group. *, *P *= 0.038 for the change at time point B vs W1 between the SVR and the TF group

### Genetic variation and molecular evolution of the HCV quasispecies during treatment in patients with different patterns of response

**Figure 2:**
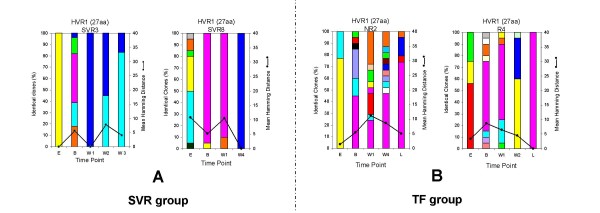
**Evolution of QC and QD within the 27 aa of the HVR1 in both group of patients**. (A) 2 representative individuals within the SVR group, SVR3 and SVR8. (B) 2 representative individuals within the TF group, 1 non-responder (NR2) and 1 relapser (R4). The vertical bars indicate the number and the proportion of viral variants within each sample. Within the vertical bars, each variant is represented by a different colour. The dominant viral strain found in each patient at Baseline is in pink colour. The other strains are represented by different colours. The same colour indicates identity between viral strain present at different time point but not between different patients. The black line indicates the quasispecies diversity calculated by the mean Hamming distance (HD) from each sample.

Samples in the SVR group showed a decrease in HD, GD, Sn-nt and Sn-aa between the B sample and the other serial samples available for analysis but none of the difference were significant (Table 2). These variations were associated with a significant reduction of HCV viral load (*P *= 0.01, Table 2). In the majority of SVR patients these changes occurred before week 2, leading to a collapse of QD followed by a decrease of viral RNA below the lower level of detection (LOD, 10 IU/mL) (Figure 2A and Table 1). Within the TF group, despite a decrease in viral load over time, this variation was not significant (*P *= 0.057, Table 2). For the TF patients who had an end of treatment response followed by relapse, genetic diversity decreased at a slower rate than within the SVR group, leading to an almost homogeneous HCV quasispecies population at the time of relapse only (R4, Figure 2B). The reduction in Sn-aa at time point B versus W1 was significant when compared the two groups of patients (*P *= 0.038, Table 2). Among the TF group, NR2 who did not response to therapy had a viral load that was stable during the course of treatment (mean 5.15 ± 0.33). In NR2 the genetic diversity increased in the first 2 weeks of treatment and then decreased slightly over the 24 weeks of treatment where samples were available (NR2, Figure 2B). The same analysis was performed on the 62 predicted aa sequence outside the HVR1 located at the 5'end of the HVR1. In all patient groups, the normalized Shannon entropy at both nucleotides and amino acids level, the genetic diversity and the genetic distance did not show any significant variation over time (*P *> 0.05, Table 2).

The analysis of individual viral variants within a patient was performed by examination of the 27 aa HVR1 sequences at each time point and grouped according to the pattern of response to therapy (Figure 2). The two representative examples of the SVR group, patient SVR3 and SVR8 depicted in Figure 2A, showed clearly that the number of viral strains present at baseline and at week 1 is reduced or retain at a low level of heterogeneity. In all SVR samples the dominant strain at week 1 of therapy represents an average of 90% of the total viral population. Interestingly, the dominant strain present at baseline was still present in 3 patients in the SVR group at week 1 (Figure 2A, SRV8 is a representative example, other results not shown) and retained dominance in two of them while disappearing in 1 patient (results not shown). In the case of the superfast responder, SFR (SVR12, Table 1), there was 100% homogeneity at the amino acid level at baseline (data not shown).

The two representative examples in TF group, patient NR2 and R4 depicted in Figure 2B, showed clearly that the number of viral strains present at baseline and at week 1 is higher than in the SVR group. Interestingly, the difference observed between the two groups was significant at both time points. At B time point in the TF group, the number of clonotypes was 6 versus 3 in the SVR group with a *P *value of 0.024, whereas at W1 time point, the number of clonotypes in the TF group was 5 versus 2 within the SVR group with a *P *value of 0.03. In all TF cases at least one strain present at B time point was retained during the course of therapy and after the end of treatment. In all TF cases at the L time point, where sample was available, the pre-dominant strain was either the dominant strain or a minor strain already present at B time point. This finding suggests the pre-existence of a "future" high fitness strain able to persist and effectively dominate the quasispecies population under interferon base anti-viral therapy.

### Phylogenetic analysis of the HCV quasispecies prior and during treatment in patient with different patterns of response

**Figure 3:**
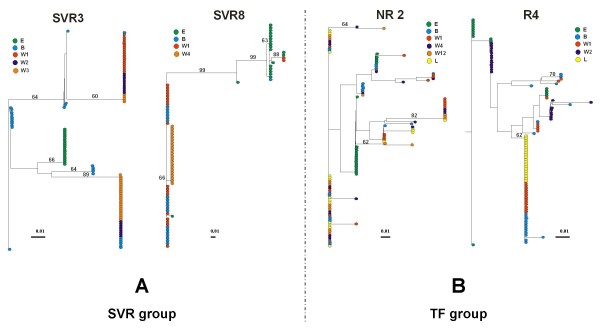
**Phylogenetic trees of all viral HVR1 amino acid sequences within each group of patients**. (A) 2 representative individuals within the SVR group, SVR3 and SVR8. (B) 2 representative individuals within the TF group, 1 non-responder (NR2) and 1 relapser (R4). The phylogenetic trees were constructed with the NEIGHBOR program in the PHYLIP package based on Kimura's distance, shown as scale bar below each tree. A bootstrap analysis using 1000 bootstrap replicates was performed to assess the reliability of each branch point. Bootstrap scores are given as percentage value. The values greater than 60% are annotated at appropriate branches. Each dot represents an individual clone. Each colour corresponds to a different time point.

To monitor viral variation and evolutionary relationships over time, phylogenetic analysis of all amino acid viral sequences of the HVR1 within a patient were performed. The phylogenetic trees represented in Figure 3 correspond to representative patterns according to therapy outcome. In the SVR group a distinct cluster of a monophyletic population was observed at E time point in 5 over 6 patients (representative example SVR3, Figure 3A) supported by a bootstrap proportion of greater than 650 of 1000 bootstrap replicates annotated at the appropriate branches as a percentage value (Figure 3A). During the course of therapy in all cases examined, the viral sequences showed distinctive clustering within the sampling time points for the SVR group. This phenomenon was not observed for the TF group. Thus for SVR patients, there was a progressive shift in the viral population over time (Figure 3A). This observation is consistent with the low level of quasispecies diversity observed during the pre-treatment assessment period and with the decrease of QD observed over time within the SVR group. In contrast, no cluster of a monophyletic population was observed at E time point within the TF group and in most cases the viral sequences showed no emergence of a cluster within the sampling time points, during the course of treatment. The NR2 case in Figure 3B is a representative example of this pattern showing intermingling of variants. This observation suggests a relative evolutionary stasis of the viral population in response to interferon based therapy compared to the pattern observed in the SVR group. However, in relapse patients a tendency to form clusters was observed at the time of relapse only, case R4 in Figure 3B. These results are consistent with the high level of QD observed within the TF group during the pre-treatment assessment period and with the decrease in QD observed in relapse patient at the time of relapse.

### Intra-sample and inter-sample genetic distance variability during treatment in patient with different patterns of response

The intra-sample analysis which is a pairwise comparison between all sequences within a particular quasispecies population, measured the level of diversity within each set of quasispecies population. At the HVR1, the mean intra-sample genetic distance variability showed no marked change over time within the SVR group (*P *> 0.05, Table 3). Within the TF group, the mean intra-sample genetic distance variability showed a slight decrease over time but the magnitude of change between the different time points were not significant (*P *> 0.05, Table 3). Overall, these results are concordant with the lower QC and QD observed within the SVR group when compared to the TF group during the pre-treatment assessment period and during the course of therapy (Figure 2).

**Table 3:** Intra- and intersample genetic variability of the HVR1 and outside the HVR1 over time in the two groups of patients

**Region**	**Patient Group**	**Samples**	^a^**Intrasample variability**				**Samples**	^b^**Intersample variability**			
					
			***Ks***	***Ka***	***Ka/Ks***	**gd**		***Ks***	***Ka***	***Ka/Ks***	**gd**
	**SVR**	**E**	0.0195 ± 0.0141	0.0308 ± 0.0130	0.998	0.0283 ± 0.0095					
		**B**	0.0236 ± 0.0175	0.0225 ± 0.0115*	**0.850**^†^	0.0233 ± 0.0091	**B-B**	0.0236 ± 0.0175	0.0225 ± 0.0115*	0.850	0.0233 ± 0.0091
		**W1**	0.0277 ± 0.0152	0.0302 ± 0.0100	**1.067**^‡^	0.0302 ± 0.0087	**B-W1**	0.0293 ± 0.0160	0.0305 ± 0.0110	1.041	0.0305 ± 0.0095
		**W2**	0.0050 ± 0.0025	0.0375 ± 0.0155	2.700	0.0280 ± 0.0125	**B-W2**	0.0070 ± 0.0045	0.0370 ± 0.0205	7.300	0.0285 ± 0.0125
		**W3/W4**	0.0000 ± 0.0000	0.0135 ± 0.0075	NA	0.0095 ± 0.0045	**B-W3/4**	0.0015 ± 0.0015	0.0150 ± 0.0085	9.333	0.0110 ± 0.0055
**HVR1**											
	**NR**	**E**	0.0292 ± 0.0185	0.0585 ± 0.0207	1.860	0.0492 ± 0.0145					
		**B**	0.0345 ± 0.0192	0.0802 ± 0.0227*	**2.427**^†^	0.0667 ± 0.0185	**B-B**	0.0345 ± 0.0192	0.0802 ± 0.0227*	2.427	0.0667 ± 0.0185
		**W1**	0.0172 ± 0.0115	0.0507 ± 0.0175	**2.033**^‡^	0.0412 ± 0.0135	**B-W1**	0.0218 ± 0.0125	0.0538 ± 0.0178	2.332	0.0432 ± 0.0130
		**W2/4**	0.0205 ± 0.0175	0.0320 ± 0.0135	1.574	0.0290 ± 0.0115	**B-W2/4**	0.0200 ± 0.0100	0.0325 ± 0.0135	1.648	0.0295 ± 0.0120
		**L**	0.0160 ± 0.0033	0.008 ± 0.0036	0.285	0.0106 ± 0.0043	**B-L**	0.0153 ± 0.008	0.0133 ± 0.0057	0.873	0.0137 ± 0.0047

**Outside**											
	**SVR**	**E**	0.0106 ± 0.005	0.0023 ± 0.0015	0.216	0.0045 ± 0.0016					
		**B**	0.0130 ± 0.0061	0.0036 ± 0.0020	0.277	0.0061 ± 0.0023	**B-B**	0.0130 ± 0.0061	0.0036 ± 0.0020	0.277	0.0061 ± 0.0023
		**W1**	0.0052 ± 0.0022	0.0017 ± 0.0015	0.327	0.0027 ± 0.0012	**B-W1**	0.0050 ± 0.0020	0.0018 ± 0.0018	0.360	0.0025 ± 0.0013
		**W2**	0.0035 ± 0.0025	0.0025 ± 0.0015	0.714	0.0025 ± 0.0020	**B-W2**	0.0035 ± 0.0025	0.0025 ± 0.0020	0.714	0.0025 ± 0.0020
		**W3/W4**	0.0015 ± 0.0015	0.0010 ± 0.0010	0.667	0.0010 ± 0.0010	**B-W3/4**	0.0015 ± 0.0015	0.0010 ± 0.0010	0.667	0.0010 ± 0.0010
	**NR**	**E**	0.0612 ± 0.0205	0.0042 ± 0.0025	0.068	0.0180 ± 0.0052					
		**B**	0.0280 ± 0.0115	0.0017 ± 0.0012	0.061	0.0085 ± 0.0032	**B-B**	0.0280 ± 0.0115	0.0017 ± 0.0012	0.061	0.0085 ± 0.0032
		**W1**	0.0132 ± 0.0085	0.0005 ± 0.0005	0.038	0.0040 ± 0.0022	**B-W1**	0.0148 ± 0.0090	0.0008 ± 0.0008	0.054	0.0043 ± 0.0025
		**W2/4**	0.0100 ± 0.0060	0.0015 ± 0.0010	0.150	0.0047 ± 0.0026	**B-W2/4**	0.0095 ± 0.0060	0.0015 ± 0.0010	0.158	0.0035 ± 0.0020
		**L**	0.0133 ± 0.0060	0.0006 ± 0.0006	0.045	0.0030 ± 0.0020	**B-L**	0.0140 ± 0.0057	0.0010 ± 0.0010	0.071	0.0043 ± 0.0020

^a ^The average number of nucleotide substitutions per nonsynonymous site and per synonymous site for all pairwise comparisons within each sampling point. ^b ^The average number of nucleotide substitutions per nonsynonymous site and per synonymous site for all pairwise comparisons for consensus of baseline for baseline sample (B-B) and follow-up samples (B-W1-2-3/4 and B-L). *Ka/Ks *indicate the ratio of nonsynonymous to synonymous nucleotide substitutions. All data represent mean ± SEM. The statistical significance of comparisons among individual samples or between the two groups of patients were analysed with non parametric Mann-Whitney U test.*, *P *= 0.05 for comparison between the two groups of patient. **†**, *P *= 0.01 for comparison between the two groups of patient. **‡**, *P *= 0.05 for comparison between the two groups of patient.

Inter-sample analysis which is the comparison of the baseline sample alone versus the consensus of baseline plus follow-up samples showed a slight increase of the mean genetic distance within the SVR group (Table 3). In contrast, within the TF group, inter-sample genetic distance variability revealed a slight decrease over time (Table 3). None of these changes were significant (*P *> 0.05, Table 3). These findings are concordant with the phylogenetic analysis indicative of a relative evolutionary stasis of the viral population in response to interferon based therapy within the TF group and a dynamic change in the quasispecies population in response to interferon based therapy within the SVR group.

Intra-sample and inter-sample genetic distance variability was determined outside the HVR1 and in all groups this regional analysis showed a lower rate of genetic variability and heterogeneity over time (Table 3).

### Rate of accumulation of synonymous and nonsynonymous substitutions during treatment in patients with different patterns of response

The accumulation rates of synonymous substitutions per synonymous site (*Ks*) and nonsynonymous substitutions per nonsynonymous site (*Ka*) were compared in each group of patients to screen for positive selection in the HVR1. Table 3 shows the intra-sample accumulation rates of synonymous and nonsynonymous substitutions at each time point and inter-sample accumulation rates of synonymous and nonsynonymous substitutions when compared to the consensus of the viral sequence derived from the B time point.

At the HVR1, in both group of patients during therapy, the intra-sample rate of nonsynonynous substitution was higher than the rate of synonymous substitution indicating that HVR1 is under positive selection (ratio *Ka*/*Ks *> 1). The number of both synonymous (*Ks*) and nonsynonymous (*Ka*) substitutions over time was higher within the TF group compared to the SVR group with a significant difference observed at B time point for *Ka *(*P *= 0.025, Table 3). Furthermore, the intra-sample ratio *Ka*/*Ks *was significantly higher in the TF group when compared to the SVR group at B time point (*P *= 0.01, Table 3) and at W1 time point (*P *= 0.05, Table 3). This result is consistent with the higher intra-sample QC and QD at B time within the TF group when compared with the SVR group. No significant difference was observed between the two groups of patients for the other follow up samples probably due to the limited number of sample available (*P *> 0.05, Table 3).

Inter-sample analysis within the SVR group showed a relatively stable *Ka*, associated with a decreasing *Ks*, hence, an increase in the magnitude of the *Ka*/*Ks *ratio in response to interferon based therapy (Table 3). In contrast, inter-sample analysis within the TF group showed a concomitant decline in *Ka *and *Ks *resulting in a progressive decrease of the *Ka*/*Ks *ratio in response to interferon based therapy (Table 3). Overall, intra-sample analysis indicates that while the QC remains relatively stable over time, the actual amino acid composition changes due to nonsynonynous mutations in the SVR group likely due to enhanced positive selection in the SVR group compared to the TF group. In contrast, the intra-sample and the inter-sample substitutions outside the HVR1 were mainly synonymous in all groups of patients suggesting that this region evolved under purifying selection (Table 3).

### Sequence analysis of the molecular clones with the 12 bp insertion

A total of 30 molecular clones were found to contain a 12 bp in-frame insertion. All these molecular clones belonged to patient SVR6, a patient from the SVR group who had been examine at E and B time point only, because no viral RNA was recovered after viraffinity protocol on the W1 sample (Table 1). For this particular patient, at E time point, 50% of clones (n = 10/20) contained the 12 bp insertion encoding the following amino acids: KT**G**G (EU023503–EU023512). At B time point 100% of clones (n = 20) contained the 12 bp insertion with 2 different non-synonymous mutations compared to the original 4 aa motif. The 12 bp insertion encoded the aa sequence KT**D**G within 85% of clones (EU023525, EU023526 and EU023528–EU023542), whereas the 12 bp insertion of the remaining 15% of individual clones encoded the aa sequence KT**E**G (EU023523, EU023524 and EU023527). Interestingly, the 3 different species harbouring the insertion contained no synonymous mutations within the region sequenced. Furthermore, the 3 variants showed conservation of 3/4 aa, the aa change occurring always at the third position of this short motif. The variant with the insertion at E time point encodes for a Glycine (G) at the third position whereas the two other variants present at B time point encode for an Aspartic Acid (D) or a Glutamic Acid (E). Aspartic Acid and the Glutamic Acid are both hydrophilic, polar and negatively charged amino acids whereas Glycine is a less hydrophilic and neutral amino acid (i.e. uncharged). These differences suggest that KT**D**G and KT**E**G motifs present at B time point are more likely coding for external motifs with the potential to bind to positively-charged molecules. These findings strongly suggest that the 12 bp insertion may be an important part of the quasispecies evolution.

The HVR1 of the HCV genome in this particular quasispecies population, i.e., SVR6, likely encodes 31 aa instead of 27 aa. In fact this is not the first description of a 12 nucleotides in-frame insertion at this position. However, this is the first reported, to our knowledge, of an in-frame insertion in a genotype 3a virus. Aizaki *et al*. [52] have reported a 12 nucleotides in-frame insertion at exactly the same position, junction of the E1 and E2 regions, within a genotype 1b isolate. Only a limited number of other variants harbouring insertions of 1 to 4 amino acids without frame shift have been reported [53-57]. These insertions occurred at the same position as the insertion we described here, i.e., 5'end of the 27 aa HVR1 [52,54]) or after the first amino acid within the HVR1 [53,55-57]. Based on GenBank database sequence analysis we found no sequence identity at both nucleotide and amino acid level between our sequence and the few variants already published [52-57]. According to their recent data, Torres-Puente *et al*. argued that variability in the size of the HVR1 could affect its antigenic property and its ability to bind to cellular receptor [57]. Their results suggest a possible association between the presence of insertion and a lack of response to therapy for genotype 1b infected patients. In contrast in our study, the patient harbouring the insertion within the HVR1 had showed a sustained virological response after the end of therapy. Further studies are needed to definitively understand the contribution of these naturally occurring variant viruses to the HCV quasispecies population dynamics and their implication in the HCV life cycle and pathogenicity.

## Discussion

In this retrospective study we aimed to characterise QS evolution in chronically infected hepatitis C genotype 3a patients, (1) in the pre-treatment period and (2) during the course of standard combination anti-viral therapy. The study outlined here is the first to evaluate QS genetic evolution in a single HCV genotype 3a population. Treatment resulted in an early virological response rate of 90% (TND at week 1 to 4 of treatment), an end of treatment response rate of 90% and a sustained virological response rate of 60%. The rate of SVR reported here is slightly lower than the rate for larger studies [58] for genotype 3a patients, probably because of the limited number of samples analysed. Age, BMI and viral load were not associated with treatment outcome as previously demonstrated in larger genotype 3a population studies [49,50]. In the present study, we have described (1) temporal changes during the pre-treatment period in Sn-aa and in HD and (2) how these changes in Sn-aa and HD relate to treatment outcome. Baseline complexity was significantly lower in the SVR groups compared to the TF group (*P *= 0.019 for Sn-aa and in HD).

Our results are in broad agreement with previous studies that have investigated viral genetic parameters as possible predictive markers of treatment outcome [28,37,43]. However, our study advances these observations and further confirms the findings reported by Yeh *et al*. on a homogeneous population of HCV genotype 1b infected patients. Our data suggests that it may be possible to predict treatment outcome on the basis of QC at an earlier stage in the treatment regimen [30]. The observed variances between our study and those of Farci *et al*. and Chambers *et al*. is likely due to differences in the genotype composition of the study population, in the methodological approach and in the genetic parameters examined [28,37,43]. In the study reported here, variables were controlled to reduce the number of parameters contributing to the analysis: (1) single genotype/subtype examined, (2) evolution rates were controlled by use of intra-patient data, (3) sera was normalised to 4 log_10 _IU/mL and (4) a previously validated proof-reading DNA polymerase based PCR methodology was used [51]. This study design, in particular, the use of intra-patient versus inter-patient controls and the use of a proof reading polymerase, likely accounts for the differences in the proportion of defective or unreadable clones (0.006) seen in our study and that reported by Farci *et al*. (0.099), *P *< 10^-6 ^(data not shown) [28]. Consequently, the inferred HCV quasispecies complexity defined in our study is likely more reflective of the true quasispecies complexity in vivo.

It is widely accepted that the genotype of the infecting virus has a very large impact on treatment efficacy and the kinetics of response in terms of actual viral load. Perhaps the quasispecies dynamic also varies by according to genotype. The investigation of the molecular changes induced by an interferon based therapy in a mixed HCV genotype infected population suffers from this caveat. [28,37]. Abbate *et al*. and Yeh *et al*. have both examined a homogeneous population of HCV genotype 1b infected patients [30,43]. At baseline, Yeh *et al*. found that the quasispecies complexity at the amino acid level was significantly lower in the SVR group than in the TF group. Conversely, Abbate *et al*., despite using a homogeneous genotype population and importantly utilised a proofreading DNA polymerase protocol, did not find any significant difference between the SVR and the TF group with respect to Shannon entropy at the nucleotide level [30,43]. However, Abbate *et al*. did not present data relating to Shannon entropy at the amino acid level [43]. Chambers *et al*. in their study on HCV genotype 1a and 1b infected patients described a trend towards a greater pre-treatment amino acid complexity in the HVR1 amongst non-responders and this pattern was significantly associated with a higher likelihood of non-response [37]. However, the authors have additionally concluded that this trend could not significantly distinguish responders from non-responders based on achievement of a SVR [37]. Our study showed that a significant difference between the SVR and the TF group existed for Shannon entropy at the amino acid level but not at the nucleotide level. These latter results are consistent with Yeh *et al *[30].

The diversity, measure by the mean HD, was significantly lower in the SVR group when compare to the TF group in our study population. This result indicates that, at baseline in the SVR group, the individual viral strains are closely related to each other, as the mean HD defines the diversity among a set of sequences. Farci *et al*. did not correlate the mean HD results at baseline to the different patterns of response [28]. Therefore it is difficult to directly compare the two studies based on the mean HD parameter.

Our findings document patterns of quasispecies change in the HVR1 in a genotype 3a population in the months prior to the start of therapy. Therapy-driven changes to the quasispecies are a key viral trait in the early response to the therapeutic pressure and likely vary according to the genotype sensitivity to pegylated interferon and ribavirin. Abbate *et al*. have reported results supporting this concept, in a single genotype population, and have postulated that the evaluation of viral quasispecies at time points earlier than baseline is likely to be more informative with respect to viral evolution [43]. Collectively this information begs the following question: what mechanism could rationally explain why a low level of quasispecies complexity and diversity prior to the start of anti-viral therapy correlates with therapy induced HCV viral clearance? Replicative homeostasis may provide a mechanistic explanation [44-47].

Replicative homeostasis (RH) consists of an epicyclical regulatory mechanism which links dynamically RNA polymerase function with quasispecies phenotypic diversity resulting in the formation of stable, but reactive, replicative equilibria [47]. Experimental evidence for RH has been recently reported by Chen *et al*. in Hepatitis B virus infection [48]. In brief, RH hypothesises that a RDRP that is highly processive has a reduced replication fidelity resulting in a high intracellular concentration of mutant genomes and consequently, a mutant spectrum of proteins. This mutant protein population (out) competes with wild type forms and RNA polymerase interactions resulting in a progressive increase in RDRP fidelity. Hepatitis C has a breadth of sequence space within which mutations can be tolerated. This epicyclic variation in viral sequence space is continuously constrained by factors such as viral fitness and the totality of the host's defence systems. The normalised Shannon entropy at the amino acids level (Sn-aa) can be considered, in part, a measure of the fidelity of the RDRP. However, fidelity can be influence by other factors. High Sn-aa equates to a highly processive RDRP, which equates to a high quasispecies complexity. The potential pre-treatment efficacy of peglyated interferon based therapy is related to Sn-aa, in our study population, as evidence by the fact that the SVR group had a significantly lower Sn-aa at time point B when compared to the TF group (*P *= 0.019, Figure 1A). The normalised Shannon entropy at the amino acids level could therefore be used to predict treatment outcome before therapy has started in a genotype 3a population. The differences between the Sn-aa at the E and B time point, even within the limited sample set examined, indicated that oscillations in the Sn-aa value occur over a short period of time. These oscillations are of limited variance for the SVR group with a trend towards reduced Sn-aa (-0.026, Table 2) and according to RH likely to be in a phase where RDRP fidelity is high [47]. Knowledge of the Sn-aa may assist in the pre-treatment identification of the approximate 20% of HCV 3a patients who will not respond to pegylated interferon based anti-viral therapy.

The separation of SVR from TF based on Sn-aa and HD suggests that real time mapping of QC and QD may identify windows of reduced Sn-aa and HD and by association, windows of enhanced treatment efficacy. Two cases that highlight the possible existence of windows of enhanced treatment efficacy are (1) SVR8 and, (2) NR2 (Figure 3): (1) SVR8 exhibited a Sn-aa and HD for the E sample that were considerably higher than that recored for the B sample, 0.467 versus 0.067, and, 10.80 versus 5.20, respectively. While it is impossible to predict what the treatment outcome would have been at time point E, the quasispecies complexity at time point B is less, existing primarily as a single strain representing 95% of clones recovered. Based on the Sn-aa, the extent of clonotype diversity and the HD, perhaps the timing of treatment of SVR8 was fortuitous. (2) Conversely, NR2 may have had a window of greater efficacy 24 weeks prior to the initiation of therapy. Specifically, the Sn-aa and HD for NR2 between the E and B time points were 0.173 versus 0.481, and, 1.40 versus 5.40, respectively. The expansion of the viable sequence space for the pre-treatment B sample correlates with reduced treatment efficacy.

The SFR (SVR12) represents an extreme example of RDRP fidelity which results in a collapsing of the quasispecies diversity at the HVR1 and likely viable sequence space. The addition of exogenous pegylated interferon and ribavirin further restricts the viable sequence space and in combination with a RDRP of high fidelity results in viral extinction.

## Conclusion

In conclusion, low Sn-aa and low HD at baseline are significantly associated with the clearance of HCV in this genotype 3a population. The replicative homeostasis hypothesis provides a probable mechanistic explanation for our findings [30,47,48]. Temporal windows of enhanced efficacy for pegylated-interferon based therapy may exist, although this will require prospective evaluation.

## Methods

### Patients

Ten patients with a chronic HCV genotype 3a infection (7 females and 3 men, mean age of 41 ± 9 years) were included in the present retrospective study. All the patients had been treated with standard pegylated α-2a interferon plus ribavirin for 24 weeks and 9/10 patients were treatment naïve at the start of the therapy (Table 1). Previously, patient SVR9 had been treated with Interferon A alone for a period of 3 months. SVR9 was off treatment for 6.5 years before the start of the standard pegylated α-2a interferon plus ribavirin treatment course. All HCV viral load measurements were determined by use of commercial assay Ampliprep/COBAS-TaqMan 48 platform (Roche Diagnostic, UK) (Table 1). Treatment outcome was defined by viral status six months post-cessation of therapy, i.e., non detectable viral RNA equated to a sustained virological response (SVR) and presence of detectable viral RNA equated to a treatment failure (TF). For the purpose of this study, sera samples were classified in (1) SVR or (2) TF in accordance to their treatment outcome (Table 1). A waiver of consent was provided by Clinical Research Ethics Committee of the Cork Teaching Hospitals as samples used in this study were surplus to requirements following diagnostic investigations.

### Design of the study

The number of viral variants, the genetic distance among the different variants (genetic diversity), the level of complexity (Shannon entropy), the evolution of HCV quasispecies and the level of viral replication were studied in serial serum samples obtained at different time points before and during the course of therapy.

All serum samples were normalised to 4 log_10 _IU/mL before RNA preparation in order to (1) standardise amplification efficiencies for intra and inter-patients sera and (2) reduce the number of variables relative to the study. The lower limit of detection (LOD) of the HVR1 RT-PCR reaction was 3 log_10 _IU/mL. Viral load 4 log_10 _IU/mL was chosen as the normalisation point for all samples. Viraffinity reagent (Biotech Support Group, US), allows the capture and the subsequent recovery of whole infectious virions, viral components, and sample preparation for subsequent detection and analysis. In the present study, 7 serum samples which had RNA level between 3–4 log_10 _IU/mL were treated with the Viraffinity™ reagent (Table 1).

For each patient the following serial serum samples were obtained: one sample between 6 to 12 months before the start of treatment (hence E, for early), one sample at day 0 before the start of treatment (hence B, for baseline) and one at week 1 of treatment (hence W1) (Table 1). Additional serial serum samples were analysed, according to the pattern of response, between week 2 and week 12 of treatment (hence, W2–W12) (Table 1). A later sample (hence, L) was analysed for the TF group (Table 1). In relapse patients, the later sample was taken at time of relapse, between 2 to12 weeks after the cessation of therapy and in the non-responder patient it was taken 3 weeks after the end of treatment (Table 1).

### Amplification of the E1/E2 region encompassing the HVR1

All serum was normalised to 4 log_10 _IU/mL by dilution in buffer Tris-Hcl 10 mM pH 7.5. Total RNA was extracted from 140 μl of the normalised sera (QIAmp Viral RNA Mini kit, Qiagen, UK) and eluted in 60 μl of molecular biology grade water. For the 7 serum samples which had RNA level between 3 log_10 _(LOD of RT-PCR reaction) and 4 log_10 _IU/mL, 1 ml of pure serum was processed in presence of 250 μl Viraffinity™ reagent (Biotech Support Group, US) according to the manufacturer recommendations and viral particles when recovered at the last round of centrifugation were directly lysed into 560 μl of lysis buffer provided in the QIAmp Viral RNA Mini kit (Qiagen, UK). Unfortunately, 4 samples had insufficient RNA to permit amplification even after Viraffinity treatment and were therefore excluded from the study (Table 1).

0.5 μg random primers mix (Promega, Madison, WI) was added to 11 μl of RNA. The RNA and primer mixture was heated at 75°C for 10 min. and then cooled on wet ice. To this was added 400 μM dNTPs (Roche, UK), 40 units RNAse inhibitor (Promega, Madison, WI), 4 μl of AMV RT 5× reaction buffer and 10 units of AMV reverse transcriptase (Promega, Madison, WI) to a final volume of 20 μl. The reaction was incubated at 42°C for 60 min. with a final 94°C, 3 min. enzyme denaturation step. The amplification of E1/E2 region encompassing the HVR1 was carried out by use of nested primers, hence, set I previously described by Ju Lin *et al *resulting in a 320 bp fragment extending from nucleotides 1254 to 1572 according to reference strain HCVCENS1 genotype 3a (GenBank accession no X76918)[59]. The primer sequences were as follow (5' to 3'): outer forward, OF (I), ATGGCATGGGATATGAT; outer reverse, OR (I), AAGGCCGTCCTGTTGA; inner forward, IF (I), GCATGGGATATGATGATGAA; inner reverse, IR (I), GTCCTGTTGATGTGCCA. The PCR reactions were performed with the proofreading *Pwo *DNA polymerase (Roche Molecular Biochemicals, UK) to ensure the accuracy of observed quasispecies diversity as previously described by Mullan *et al*. [51]. First round-PCR was performed by mixing 5 μl of RT reaction mixture to a final volume of 50 μl containing 15 pmol each of OF (I) and OR (I) primers, 200 μM dNTP mix, 5 μl 10× *Pwo *PCR MgSO_4 _free buffer (Roche, UK), 1.5 mM MgSO_4 _and 2.5 units of *Pwo *DNA Polymerase. Samples were amplified in a GeneAmp PCR System 2700 thermal cycler (Perkin Elmer, Kenilworth, NJ) under the following thermal cycling profile: 3 min. at 94°C for initial denaturation of cDNA; 35 cycles of 94°C, 15 s; 51°C, 30 s and 72°C, 45 s; followed by final elongation at 72°C for 7 min. The secondary nested PCR reaction was done using 4 μl of primary PCR product as a template and identical composition to the first round of PCR, except for the relevant nested primer set, IF (I) and IR (I) (15 pmol each), a MgSO_4 _concentration adjustment to 1 mM and a melting temperature (Tm) of 53°C for the annealing step.

Suitable precautions were taken to reduce the risk of inter-sample contamination as suggested by Kwok and Higuchi [60]. In addition, for each test sample, a negative control was analysed in parallel throughout the entire procedure. To screen for potential contamination of product DNA, the viral sequences were analysed by cross comparisons of all PCR product sequences included in the study as well as comparisons with other viral sequences generated in the laboratory by using a Neighbor-Joining algorithm (PHYLIP) [61].

### Molecular cloning and sequencing

After gel purification (Qiaquick Gel Extraction Kit, QIAGEN, UK), the amplicons from E1/E2 were cloned into pCR4 Blunt-Topo vector by use of the Zero Blunt TOPO system (Invitrogen, Belgium) and transformed into chemically competent *Escherichia Coli *strain TOP10 (Invitrogen, Belgium). This cloning kit provides a quick and highly efficient one-step cloning strategy for direct insertion of blunt-end PCR products, generated by thermostable proof reading polymerases such as Pwo DNA polymerase, into a plasmid vector specifically designed for sequencing. Transformants were detected and 20 recombinant clones for each sample were selected at random, plasmid DNA was purified by use of QIAprep Spin Miniprep Kit (Qiagen, UK) and screened for presence of inserts. The double-stranded plasmid DNA sequencing was out sourced to MWG-Biotech, Germany. Where sequences had a high degree of homogeneity primary and secondary PCR amplicons were re-generated in duplicate from the initial cDNA template. After cloning into pCR4 Blunt-Topo vector only 5 additional clones were recovered for each new generated amplicon, purified and sequenced as previously described.

### GenBank accession numbers

The sequences reported in this study have been assigned the following GenBank accession nos (EU023073–EU023906) .

### Sequence analysis of the E1/E2 region encompassing the HVR1

Sequence similarities between the sequences generated during this study were examined by use of the BLASTN web program  or .

Sequence alignments were performed with CLUSTALW (version 1.74)  as previously described [62]. Sequence analysis was performed after exclusion of all the defective sequences due to nucleotide insertion or deletion or mutation producing a stop codon. Genetic diversity was calculated by analysis of predicted amino acid sequences amplified from E1 and E2 genes of the HCV genome including and excluding the 27 aa of the HVR1. The genetic diversity was calculated as Hamming distance, or (1-S) ×100, where S is the fraction of shared sites in two aligned nucleotide sequences. The mean Hamming distance which is the average of the values taken for all sequence pairs derived from a single sample was separately calculated within the HVR1 (27 aa) and on the sequence outside the HVR1 (62 aa) [28]. The mean genetic distance (GD), the number of synonymous (silent) nucleotide substitutions per synonymous site (*Ks*) and the number of nonsynonymous (amino acid replacement) nucleotide substitutions per nonsynonymous site (*Ka*) were calculated with the Kimura two-parameter method, all sites, in the Molecular Evolutionary Genetics Analysis software package (MEGA2 program, [63]. The average number of *Ks *and the average number of *Ka *relative to the ancestral consensus sequence were calculated for each time point within a single patient with the MEGA2 program. Sequences obtained from each time point were compared with the consensus reference sequence of the baseline sample [28,37].

The complexity of the HCV strain in the region of interest was quantified by calculating the normalised Shannon entropy (Sn) at both nucleotides and amino acids level. The Shannon entropy is a measure of the proportion of identical sequences in a mutant distribution. The possible values of Sn range from zero (when all genomes are identical) to one (when all genomes differ from one another). Sn was calculated following the formula: Sn = Σ_i_[(*p*_i _× ln *p*_i_)ln n], in which *p*_i _is the frequency of each sequences in the mutant spectrum and n is the total number of sequences compared [64]. Phylogenetic analyses were conducted by using the NEIGHBOR program in the PHYLIP package [65], as previously described [62].

### Statistical analysis

The results are expressed as the mean ± SE. The statistical significance of comparisons among individual samples or between the two groups of patients were analysed with non parametric Mann-Whitney U test. A Chi Square Test was performed to compare the overall proportion of viable sequences obtained in our study and in Farci *et al*. study [28]. In all tests a *P *value less than 0.05 was considered statistically significant.

## Competing interests

The authors declare that they have no competing interests.

## Authors' contributions

IM contributed to the experimental design, sequence alignments, data analysis and preparation of manuscript. JL determined qualitative, quantitative and genotype of clinical specimens described here. OC and EKW are clinicians who manage HCV at Cork University Hospital. LF supervised the project and assisted with analysis and preparation of manuscript.
